# Associations Between Leisure-Time Physical Activity Level and Peripheral Immune Cell Populations in the US General Population, Analysis of the National Health and Nutrition Examination Survey Data, 1999–2018

**DOI:** 10.1186/s40798-023-00643-y

**Published:** 2023-10-28

**Authors:** Dan Lin, Cheryl L. Thompson, Djibril M. Ba, Joshua E. Muscat, Shouhao Zhou, Connie J. Rogers, Kathleen M. Sturgeon

**Affiliations:** 1 Department of Public Health Sciences, Penn State Cancer Institute, Pennsylvania State University College of Medicine, CH69, 500 University Drive, Hershey, PA 17033 USA; 2 Department of Nutritional Sciences, University of Georgia College of Family and Consumer Sciences, Athens, GA 30602 USA

**Keywords:** Physical activity, Leukocytes, Blood platelets, Inflammation, Exercise immunology

## Abstract

**Background:**

Chronic levels of inflammation are associated with higher risk of many chronic diseases. Physical activity (PA) lowers the risk of cancer, cardiovascular disease (CVD), diabetes and others. One mechanism for PA-induced protection may be through the immune system. We investigated the association between leisure-time PA and peripheral immune cell populations in a large nationally representative sample of the US general population.

**Methods:**

A total of 17,093 participants [mean (SE) age of 41.6 (0.3) years] of the National Health and Nutrition Examination Survey 1999–2018 were included. Self-reported leisure-time PA was converted to metabolic equivalent of task hours per week (MET-hrs/wk). White blood cell (WBC) count, WBC ratios, and platelet count were derived. Multivariable linear regression analyses were used to estimate associations between leisure-time PA level and peripheral immune cell populations. Multivariable logistic regression analyses were used to estimate associations between leisure-time PA and metrics of WBC count and neutrophil-to-lymphocyte ratio (NLR) which may predict mortality.

**Results:**

A higher leisure-time PA level was associated with a lower WBC count (> 14.0 vs. < 1.2 MET-hrs/wk adjusted mean (95% confidence interval [CI]): 7.12 (6.86, 7.38) vs. 7.38 (7.12, 7.64) 1000 cells/μL, *P*_trend_ < 0.001) and a lower NLR (> 14.0 vs. < 1.2 MET-hrs/wk adjusted mean (95% CI) 2.04 (1.90, 2.18) vs. 2.13 (1.99, 2.28), *P*_trend_ = 0.007). Leisure-time PA level was not associated with lymphocyte-to-monocyte ratio (LMR; *P*_trend_ = 0.25) or platelet-to-lymphocyte ratio (PLR; *P*_trend_ = 0.69). Compared to the lowest leisure-time PA level (< 1.2 MET-hrs/wk), the highest leisure-time PA level (≥ 14.0 MET-hrs/wk) was associated with a lower probability of a high WBC count (> 8.1 × 10^9^ cells/L; odds ratio [OR] = 0.76, 95% CI = 0.66–0.88) and high NLR (> 2.68; OR = 0.84, 95% CI = 0.72–0.99), which may predict CVD and all-cause mortality. The highest leisure-time PA level also linked to a lower probability of a high WBC count (≥ 8.3 × 10^9^ cells/L; OR = 0.76, 95% CI = 0.66–0.88), which may predict cancer mortality.

**Conclusions:**

We observed an inverse association between leisure-time PA level, WBC count, and NLR, particularly for neutrophil levels. These results suggest that participants at higher levels of leisure-time PA may have lower levels of inflammation, which may be important for future chronic disease outcomes.

**Supplementary Information:**

The online version contains supplementary material available at 10.1186/s40798-023-00643-y.

## Key Points

• In this cross-sectional analysis of 17,093 participants, a higher leisure-time physical activity level was associated with a lower white blood cell (WBC) count and a lower neutrophil-to-lymphocyte ratio (NLR).
• In gender-specific analysis, the inverse association between leisure-time physical activity level and WBC count was consistent in both genders, while the inverse relationship with NLR was observed in women exclusively.
• Participants at higher levels of leisure-time physical activity may have lower levels of inflammation, which may be important for future chronic disease outcomes.

**Supplementary Information:**

The online version contains supplementary material available at 10.1186/s40798-023-00643-y.

## Introduction

Chronic inflammation is one underlying pathway in the etiology of chronic diseases, such as cardiovascular disease (CVD) and cancer [1–3]. Inflammation involves white blood cells (WBCs, e.g., neutrophils, lymphocytes, and monocytes) [2, 4]. Another important component of an inflammatory response is platelets [5]. Peripheral immune cell populations are easily accessible through complete blood counts (CBCs), a blood test commonly used in the clinic. Due to their accessibility, WBC count, platelet count, and clinically relevant ratios (neutrophil-to-lymphocyte ratio (NLR), lymphocyte-to-monocyte ratio (LMR), and platelet-to-lymphocyte ratio (PLR)) are widely used to assess the role of inflammation in the development of chronic diseases [1, 5–7].

WBC count, NLR, LMR, and PLR are predictors of chronic diseases and mortality in diverse populations [8–12]. For example, a higher WBC count was associated with a higher risk of coronary heart disease, stroke, lung cancer, and breast cancer [10, 13]. Similar positive associations between NLR and PLR and chronic diseases have been observed [8, 14]. In contrast, a lower LMR was associated with a higher risk of certain cancers [8]. In a national sample of the US population, high WBC count (> 8.1 × 10^9^ cells/L) and high NLR (> 2.68) were consistently associated with a higher risk of CVD mortality and all-cause mortality [11, 15]. In addition, a high WBC count (≥ 8.3 × 10^9^ cells/L) was associated with an increased risk of cancer mortality [12]. Thus, these thresholds in WBC count and NLR may be clinically meaningful in the US population.

Leisure-time physical activity, as a modifiable lifestyle behavior, plays an important role in reducing, or preventing chronic inflammation [16]. Leisure-time physical activity is any physical activity outside of work and may include both intensive and non-intensive activities. Previous reports have observed an association between physical activity level and specific peripheral immune cell populations in various populations [17–20]. Of total physical activity, higher levels of moderate-to-vigorous physical activity, measured objectively, were associated with a lower WBC count [20]. Additionally, high-intensity exercise training decreased NLR significantly over a 3-week intervention period [18]. However, the relationship between leisure-time physical activity and other peripheral immune cell populations, such as LMR and PLR, is unknown.

There are additional knowledge gaps in the epidemiology of physical activity and immunology. First, the association between leisure-time physical activity level and peripheral immune cell populations by sex is unclear. Differences by sex have been observed such that associations between fitness level, WBCs, and mortality are less consistent in women compared to men [21]. Second, while previous studies have observed lower WBC count with higher physical activity levels, their reports are largely limited to assessment of linear trend [17, 20]. Assessing the association between leisure-time physical activity level and metrics of WBC count and NLR which predict mortality in the US general population may be clinically meaningful.

In this study, we aimed to investigate the associations between leisure-time physical activity level and peripheral immune cell populations in a nationally representative sample of the US population. Given observations from previous studies, we hypothesized that leisure-time physical activity level would be inversely associated with peripheral immune cell populations [20, 22, 23]. Specifically, we hypothesized that higher leisure-time physical activity levels would be associated with lower WBC counts, a lower NLR, a higher LMR, and a lower PLR. Further, we also hypothesized that higher levels of leisure-time physical activity would decrease the probability of WBC count and NLR above predictive thresholds which may predict disease-specific and overall mortality, in comparison to the lowest leisure-time physical activity level.

## Methods

### Study Population

The study population consisted of participants in the National Health and Nutrition Examination Survey (NHANES), 1999–2018. The NHANES is a cross-sectional study that uses a complex, multistage probability design to obtain a nationally representative sample of the US civilian noninstitutionalized population. The survey administered household interviews for lifestyle information and health conditions and conducted examinations including anthropometric assessments in mobile examination centers (MECs). More details are available on the NHANES website [24].

Of participants aged 18 years or older in NHANES 1999–2018, we excluded participants who (1) had missing data on questionnaire-based physical activity, (2) fasted less than nine hours prior to blood collection, (3) had missing data on peripheral blood lymphocyte counts, neutrophil counts, monocyte counts, and platelet counts, (4) had extreme daily energy intake (< 800 kcal or > 4200 kcal for men and < 500 kcal or > 3500 kcal for women) [25], (5) had a self-reported history or unknown history of diabetes, CVD, or cancers, and (6) had missing data on any covariate information. The final population included 17,093 participants for the analysis in the present study.

### Leisure-Time Physical Activity Assessment

Leisure-time physical activity level was assessed by two interviewer-administered physical activity questionnaires. Participants in the NHANES 1999–2006 cycles were asked about the duration and frequency of moderate and vigorous levels of physical activity during their leisure time over the past 30 days. In 2007, NHANES adopted another physical activity questionnaire which was based on the Global Physical Activity Questionnaire [26]. Participants in NHANES 2007–2018 cycles were asked about the duration and frequency of moderate and vigorous recreational physical activity in a typical week. In all cycles, participants were provided examples of moderate activities (e.g., brisk walking, golf, and dancing) and vigorous activities (e.g., aerobics, running, and lap swimming). The duration of leisure-time physical activity at each level (moderate and vigorous) was calculated as hours per week (hrs/wk). Metabolic equivalent of tasks (METs) were assigned as a score of 4.0 for moderate and a score of 8.0 for vigorous leisure-time physical activity levels according to the NHANES guidelines [26]. The intensity of physical activity is quantified relative to the average amount of energy expended at rest, as determined by oxygen consumption. Average energy expenditure in the resting state is approximately 3.5 ml/kg/min of oxygen, this equals 1 MET. A 4 MET physical activity uses approximately 4 times the amount of oxygen at rest (14 ml/kg/min). The average MET-hrs/wk for leisure-time physical activity was calculated for participants in NHANES 1999–2006, based on physical activity level in the past 30 days. The total MET-hrs/wk for leisure-time physical activity was computed for participants in NHANES 2007–2018, based on physical activity level in a typical week.

### Complete Blood Count

Prior to blood collection, a fasting questionnaire was administered to determine the length of fast and the time of venipuncture for participants. Blood samples from each participant were collected by a certified medical technologist or phlebotomist. The methods used to derive CBC parameters were based on the Beckman Coulter method of counting and sizing, in combination with an automatic diluting and mixing device for sample processing, and a single beam photometer for hemoglobinometry [27]. Total WBC, neutrophil, lymphocyte, monocyte, and platelet counts were derived. NLR, LMR, and PLR were calculated as ratios between the neutrophil and lymphocyte counts, lymphocyte and monocyte counts, and platelet and lymphocyte counts, respectively.

### Predictive Thresholds of WBC Count and NLR

Two thresholds were adopted to categorize predictive WBC count. First, a WBC count > 8.1 × 10^9^ cells/L is associated with higher risks of CVD and all-cause mortality in the NHANES population [11]. Second, a WBC count ≥ 8.3 × 10^9^ cells/L is associated with a higher risk of cancer mortality according to another report from NHANES [12]. Similarly, one threshold was adopted to categorize predictive NLR: NLR > 2.68 is associated with significantly higher risks of CVD and all-cause mortality in the NHANES population [15].

### Covariates

Covariates included variables known to be associated with peripheral immune cell population levels based on existing literature [19, 20, 28]. Age (years), gender (men and women), BMI (kg/m^2^, < 18.5, 18.5 to < 22.5, 22.5 to < 25, 25 to < 30, and ≥ 30), race/ethnicity (Mexican American, Non-Hispanic White, Non-Hispanic Black, and other), family income (low, middle, and high), education level (high school or less, some college, and college or more), marital status (married/living with a partner, never married/single, and separated/divorced/widowed), smoking status (never, former, and current smokers), alcohol intake (g/d, nondrinker, > 0 to < 15, and ≥ 15), daily energy intake (kcal/day), time of venipuncture (morning, afternoon, and evening), and history of arthritis (yes and no) were included. Survey year (1999–2006, and 2007–2018) was additionally adjusted as a covariate in our analysis because different physical activity questionnaires were used in 1999–2006 and 2007–2018. Menopausal status (premenopausal, surgical menopause, and natural menopause) and female hormone use (yes and no) were additionally adjusted for analysis in women. Income level was measured by a family poverty income ratio (PIR). The PIR is based on a comparison of family income with the poverty threshold determined by the U.S. Bureau of Census [29]. Ratios of < 1 are considered below the poverty line. The family income was categorized as: low (PIR ≤ 130% of the poverty line), middle (PIR > 130% but ≤ 350% of the poverty line), and high (PIR > 350% of the poverty line) [29].

### Statistical Analysis

Descriptive statistics were computed for study variables. Characteristics were presented as the weighted mean and compared using analysis of variance (ANOVA) for continuous variables, or weighted prevalence and compared using Chi-square test for categorical variables. NHANES provided weights to ensure the representative and unbiased estimation of the total civilian noninstitutionalized adult US population. Leisure-time physical activity level was categorized into quartiles based on the distribution of MET-hrs/wk among all eligible participants. Linear regression analyses (proc surveyreg; SAS institute) were used to estimate adjusted means of peripheral immune cell populations (continuous) with 95% confidence intervals (CIs) across leisure-time physical activity levels. The first regression model (Model 1) was adjusted for age at the interview, gender, and survey year. The multivariable-adjusted model (Model 2) was additionally adjusted for BMI, race/ethnicity, family income, education, marital status, current smoking status, alcohol intake, daily energy intake, menopausal status, female hormone use, time of venipuncture, and history of arthritis. Trend tests were conducted by using the median value for each physical activity category as a continuous variable in regression [30].

Multivariable logistic regression models (proc surveylogistic; SAS institute) were used to evaluate the associations between leisure-time physical activity level and thresholds of WBC count and NLR which predict mortality, adjusted by the same covariates in linear regression models. Odds ratios (ORs) with 95% CIs were calculated to demonstrate the strength and direction of each association. Because NHANES adopted another physical activity questionnaire in 2007, we conducted a sensitivity analysis for participants in 2007–2018 cycles to determine if the association in this sub-population was consistent with the total population. All statistical analyses were conducted in SAS 9.4 (Cary, NC). Statistical significance was assessed at an alpha level of 0.05.

## Results

Characteristics by leisure-time physical activity level in our population were described (Table 1) in total and by quartiles of physical activity level (quartile 1 =  < 1.2; quartile 2 = 1.2 to < 5.2; quartile 3 = 5.2 to < 14.0; quartile 4 =  ≥ 14.0 MET-hrs/wk). Compared to participants with the lowest level of physical activity (Q1), participants with the highest physical activity level (Q4) were younger (Q1 vs. Q4: 43.8 vs. 40.5 years), less likely to be female (52.6% vs. 45.0%), more likely to be overweight (33.3% vs. 35.3%), but less likely to be obese (36.2% vs. 25.2%), more likely to be non-Hispanic White (67.4% vs. 71.1%), less likely to have a low family income (9.8% vs. 6.6%), more likely to have college or more education (22.7% vs. 37.7%) and be single (never married: 19.7% vs. 25.4%), less likely to be current smokers (22.5% vs. 16.2%), had a higher daily energy intake (2110.9 vs. 2183.2 kcal/d), and less likely to have a history of arthritis (21.5% vs. 15.5%).

**Table 1:** Characteristics of participants, according to leisure-time physical activity level

Characteristics*	Overall *n* = 17,093	Leisure-time physical activity level, MET-hrs/wk				*p* value
		Q1	Q2	Q3	Q4	
Age, yr mean ± SE	41.6 ± 0.3	43.8 ± 0.4	40.3 ± 0.5	41.6 ± 0.4	40.5 ± 0.4	< 0.001
Gender, *n* (%)						< 0.001
Male	8363 (50.1)	2232 (47.4)	1788 (49.2)	1885 (48.9)	2458 (55.0)	
Female	8730 (49.9)	2660 (52.6)	2011 (50.8)	2082 (51.1)	1977 (45.0)	
BMI, *n* (%)						< 0.001
< 18.5 kg/m^2^	381 (2.1)	124 (2.4)	92 (2.5)	94 (2.1)	71 (1.5)	
18.5 to < 22.5 kg/m^2^	2579 (16.3)	608 (12.9)	676 (19.1)	578 (16.0)	717 (17.2)	
22.5 to < 25 kg/m^2^	2970 (18.5)	692 (15.1)	663 (19.1)	733 (19.1)	882 (20.8)	
25 to < 30 kg/m^2^	5814 (34.1)	1630 (33.3)	1257 (33.1)	1357 (34.5)	1570 (35.3)	
≥ 30 kg/m^2^	5349 (28.9)	1838 (36.2)	1111 (26.2)	1205 (28.3)	1195 (25.2)	
Race/ethnicity, *n* (%)						< 0.001
Mexican American	2734 (6.8)	869 (9.8)	581 (5.4)	602 (5.6)	682 (6.6)	
Non-Hispanic White	8615 (73.4)	2123 (67.4)	2236 (78.9)	2162 (76.3)	2094 (71.1)	
Hon-Hispanic Black	3184 (9.1)	960 (10.4)	614 (7.4)	704 (8.4)	906 (10.2)	
Others	2560 (10.7)	940 (12.4)	368 (8.3)	499 (9.8)	753 (12.1)	
Family income, *n* (%)						< 0.001
Low	2734 (6.8)	869 (9.8)	581 (5.3)	602 (5.6)	682 (6.6)	
Middle	9863 (78.4)	2652 (73.5)	2404 (82.9)	2383 (80.7)	2424 (76.6)	
High	4496 (14.8)	1371 (16.7)	814 (11.8)	982 (13.7)	1329 (16.9)	
Education level, *n* (%)						< 0.001
High school or less	6565 (32.4)	2511 (44.4)	1248 (29.3)	1297 (27.9)	1509 (28.2)	
Some college	5653 (33.8)	1468 (32.8)	1328 (34.3)	1352 (34.2)	1505 (34.1)	
College or more	4875 (33.7)	913 (22.7)	1223 (36.4)	1318 (37.9)	1421 (37.7)	
Marital status, *n* (%)						< 0.001
Married/living with partner	10,472 (64.1)	2291 (63.8)	2387 (64.4)	2445 (64.2)	2649 (64.1)	
Never married	4026 (23.1)	948 (19.7)	910 (23.9)	946 (23.3)	1222 (25.4)	
Widowed/separated/divorced	2595 (12.8)	953 (16.5)	502 (11.6)	576 (12.5)	564 (10.5)	
Alcohol intake, *n* (%)						0.14
Nondrinker	2098 (10.3)	691 (11.2)	442 (10.1)	474 (10.4)	491 (9.4)	
> 0–15 g/day	11,725 (67.3)	3379 (69.4)	2610 (66.6)	2691 (66.4)	3045 (66.7)	
> 15 g/day	3270 (22.5)	822 (19.4)	747 (23.3)	802 (23.2)	899 (23.9)	
Smoking status, *n* (%)						< 0.001
Never	10,323 (59.0)	2908 (57.9)	2285 (58.8)	2403 (59.2)	2727 (60.2)	
Former	3640 (22.4)	906 (19.5)	857 (23.0)	893 (23.3)	984 (23.6)	
Current	3130 (18.6)	1078 (22.5)	657 (18.2)	671 (17.5)	724 (16.2)	
Time of venipuncture, *n* (%)						< 0.001
Morning	14,556 (87.8)	4322 (88.0)	3120 (82.2)	3316 (83.0)	3798 (86.0)	
Afternoon	2066 (11.9)	485 (10.0)	512 (12.7)	535 (13.2)	534 (11.7)	
Evening	471 (3.3)	85 (2.0)	167 (2.8)	116 (2.3)	103 (1.8)	
Survey year, *n* (%)						< 0.001
1999–2006	9171 (59.2)	1028 (27.7)	3275 (87.7)	2844 (74.4)	2024 (47.5)	
2007–2018	7922 (40.8)	3864 (72.3)	524 (12.3)	1123 (25.6)	2411 (52.5)	
Daily energy intake, kcal/d mean ± SE	2176.8 ± 12.9	2110.9 ± 15.8	2227.9 ± 26.7	2184.8 ± 20.1	2183.2 ± 14.3	< 0.001
Ever had arthritis, *n* (%)	3030 (16.8)	1117 (21.5)	570 (14.4)	656 (15.8)	6.87 (15.5)	< 0.001

*SE* Standard error, *BMI* Body mass index, *MET* Metabolic equivalent of task, *Q* Quartile

*Characteristics are presented weighted mean and weighted prevalence

### Leisure-Time Physical Activity and Peripheral Immune Cell Populations

Associations between leisure-time physical activity level and peripheral immune cell populations in the total population are shown in Table 2. In the multivariable-adjusted model (Model 2), a higher leisure-time physical activity level was associated with a lower WBC count (Q4 vs. Q1 adjusted mean (95% CI) 7.12 (6.86, 7.38) vs. 7.38 (7.12, 7.64) 1000 cells/μL, *P*_trend_ < 0.001). Leisure-time physical activity level was inversely associated with neutrophil count (Q4 vs. Q1 adjusted mean (95% CI) 4.15 (3.95, 4.36) vs. 4.36 (4.15, 4.57) 1000 cells/μL, *P*_trend_ < 0.001) and monocyte count (Q4 vs. Q1 adjusted mean (95% CI) 0.54 (0.52, 0.57) vs. 0.56 (0.54, 0.59) 1000 cells/μL, *P*_trend_ = 0.007). Leisure-time physical activity level was not associated with lymphocyte count (*P*_trend_ = 0.37) and platelet count (*P*_trend_ = 0.11).

**Table 2:** Adjusted means (95% CI) for WBC and platelet counts by leisure-time physical activity level in total population (*N* = 17,093)

	Leisure-time physical activity level, MET-hrs/wk				*P*_trend_
	Quartile1 (*n* = 4892)	Quartile2 (*n* = 3799)	Quartile3 (*n* = 3967)	Quartile4 (*n* = 4435)	
WBC count, 1000 cells/uL, mean (95% CI)					
Model 1^a^	7.04 (6.94, 7.14)	6.65 (6.55, 6.79)	6.61 (6.52, 6.70)	6.54 (6.45, 6.62)	< 0.001
Model 2^b^	7.38 (7.12, 7.64)	7.17 (6.91, 7.42)	7.12 (6.86, 7.37)	7.12 (6.86, 7.38)	< 0.001
Neutrophil count, 1000 cells/uL, mean (95% CI)					
Model 1^a^	4.20 (4.11, 4.28)	3.92 (3.84, 4.00)	3.88 (3.80, 3.95)	3.82 (3.75, 3.89)	< 0.001
Model 2^b^	4.36 (4.15, 4.57)	4.20 (4.00, 4.40)	4.16 (3.95, 4.36)	4.15 (3.95, 4.36)	< 0.001
Lymphocyte count, 1000 cells/uL, mean (95% CI)					
Model 1^a^	2.05 (2.02, 2.07)	1.97 (1.94,2.00)	1.97 (1.94, 1.99)	1.96 (1.93, 1.99)	0.002
Model 2^b^	2.21 (2.13, 2.29)	2.18 (2.10, 2.26)	2.17 (2.09, 2.26)	2.18 (2.10, 2.27)	0.37
Monocyte count, 1000 cells/uL, mean (95% CI)					
Model 1^a^	0.55 (0.54, 0.56)	0.53 (0.51, 0.54)	0.53 (0.52, 0.54)	0.52 (0.51, 0.53)	< 0.001
Model 2^b^	0.56 (0.54, 0.59)	0.55 (0.52, 0.57)	0.55 (0.53, 0.57)	0.54 (0.52, 0.57)	0.007
Platelet count, 1000 cells/uL, mean (95% CI)					
Model 1^a^	259.42 (256.58, 262.26)	253.58 (250.33, 256.83)	254.80 (251.85, 257.75)	252.81 (250.17, 255.45)	0.016
Model 2^b^	260.88 (254.93, 266.82)	257.35 (251.20, 263.50)	258.05 (252.11, 264.00)	256.96 (251.05, 262.88)	0.11

*WBC* White blood cell, *MET* Metabolic equivalent of task, *CI* Confidence interval

^a^Adjusted for age, gender, and survey year

^b^Adjusted for age, gender, survey year, BMI, race/ethnicity, family income, education level, marital status, smoking status, alcohol intake, daily energy intake, time of the blood draw, and history of arthritis

Associations between leisure-time physical activity levels and peripheral immune cell populations in men and women were presented, respectively (Additional file 1: Tables S1, S2). Compared to the total population, we observed the following differences in the gender-specific population: (1) leisure-time physical activity was not associated with monocyte count in men (*P*_trend_ = 0.22) and women (*P*_trend_ = 0.10), and (2) leisure-time physical activity level was associated with platelet count in men (*P*_trend_ = 0.048).

Observations for the associations between leisure-time physical activity level and WBC populations count in the sub-population (participants in 2007–2018) were consistent with the full sample (data not shown). However, leisure-time physical activity level was inversely associated with platelet count in participants in 2007–2018 (*P*_trend_ = 0.003). While not common, certain conditions may affect immune cell populations. As a result, a sensitivity analysis on 16,183 participants was conducted by excluding those with a history of stroke, chronic obstructive pulmonary disease (COPD), and hepatitis. Observations in the sub-populations were consistent with the full sample (Additional file 1: Table S3).

### Leisure-Time Physical Activity and Ratios of Peripheral Immune Cell Populations

**Fig. 1:**
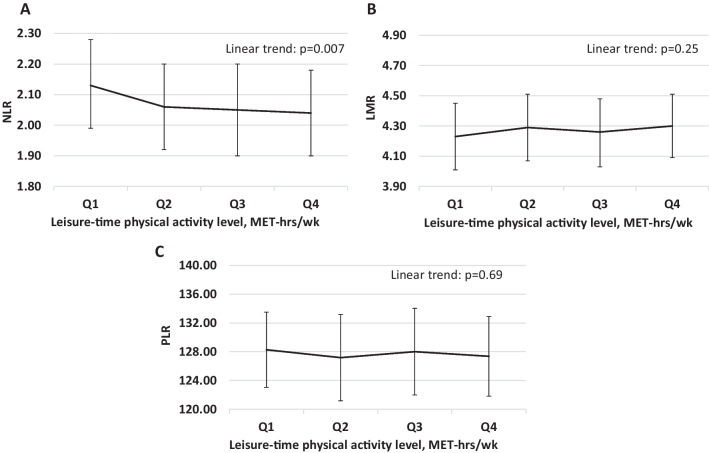
Adjusted^a^ means (95% CIs) for **A** NLR, **B** LMR, and **C** PLR, by quartiles of leisure-time physical activity. *NLR* Neutrophil-to-lymphocyte ratio, *LMR* Lymphocyte-to-monocyte ratio, *PLR* Platelet-to-lymphocyte ratio, *CI* Confidence interval, *MET* Metabolic equivalent of task, *Q* Quarter. ^a^Adjusted for age, gender, survey year, BMI, race/ethnicity, family income, education level, marital status, smoking status, alcohol intake, daily energy intake, time of the blood draw, and history of arthritis

An inverse association between leisure-time physical activity level and NLR in the total population (Model 2: Q4 vs. Q1 adjusted mean (95% CI) 2.04 (1.90, 2.18) vs. 2.13 (1.99, 2.28)) was supported by a significant linear trend (Fig. 1A; *P*_trend_ = 0.007). Leisure-time physical activity level was not associated with LMR (Fig. 1B; *P*_trend_ = 0.25) or PLR (Fig. 1C, *P*_trend_ = 0.69). The observation for NLR, LMR, and PLR in women was consistent with the total population (Additional file 1: Table S2). However, leisure-time physical activity was not associated with NLR in men (Additional file 1: Table S1; NLR: *P*_trend_ = 0.25).

### Leisure-Time Physical Activity and Thresholds of WBC Count and NLR that Predict Mortality

**Fig. 2:**
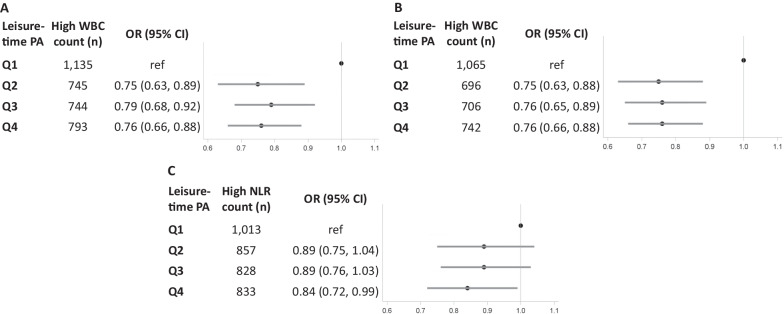
Adjusted^a^ ORs (95% CIs) for **A** high WBC count (> 8.1 × 10^9^ cells/L) relevant to CVD and all-cause mortality, **B** high WBC count (≥ 8.3 × 10^9^ cells/L) relevant to cancer mortality, and **C** high NLR (> 2.68) relevant to CVD and call-cause mortality, by quartiles of leisure-time physical activity. *OR* Odds ratio, *CI* Confidence interval, *WBC* White blood cell, *NLR* Neutrophil-to-lymphocyte ratio, *PA* Physical activity, *Q* Quarter. ^a^Adjusted for age, gender, survey year, BMI, race/ethnicity, family income, education level, marital status, smoking status, alcohol intake, daily energy intake, time of the blood draw, and history of arthritis

Compared to the lowest leisure-time physical activity level (< 1.2 MET-hrs/wk), any leisure-time physical activity level ≥ 1.2 MET-hrs/wk was associated with a lower probability of WBC count above threshold (> 8.1 × 10^9^ cells/L) that may predict CVD mortality and all-cause mortality (Fig. 2A; Q2 vs Q1: OR = 0.75, 95% CI = 0.63–0.89, Q3 vs. Q1: OR = 0.79, 95% CI = 0.68–0.92, Q4 vs. Q1: OR = 0.76, 95% CI = 0.66–0.88). In addition, compared to the lowest leisure-time physical activity level, any leisure-time physical activity level ≥ 1.2 MET-hrs/wk was associated with a lower probability of WBC count above threshold (≥ 8.3 × 10^9^ cells/L) that may predict cancer mortality (Fig. 2B; Q2 vs Q1: OR = 0.75, 95% CI = 0.63–0.88, Q3 vs. Q1: OR = 0.76, 95% CI = 0.65–0.89, Q4 vs. Q1: OR = 0.76, 95% CI = 0.66–0.88). Compared to those with the lowest leisure-time physical activity level, participants with the highest leisure-time physical activity level (≥ 14.0 MET-hrs/wk) had a lower probability of NLR above the threshold (> 2.68) that may predict CVD mortality and all-cause mortality (Fig. 2C; Q4 vs. Q1: OR = 0.84, 95% CI = 0.72–0.99).

## Discussion

Our study investigated the association between leisure-time physical activity levels and peripheral immune cell populations in the general US population. We observed that (1) a higher leisure-time physical activity level was associated with a lower WBC count and a lower NLR in the total population and women, (2) a higher level of leisure-time physical activity was associated with lower WBC and platelet counts in men, (3) leisure-time physical activity was not associated with LMR or PLR, (4) leisure-time physical activity level above 1.2 MET-hrs/wk was associated with a lower probability of WBC count above predictive thresholds which may predict CVD, cancer, and all-cause mortalities, and (5) leisure-time physical activity level above 14.0 MET-hrs/wk was associated with a lower probability of NLR above the threshold which may predict CVD and all-cause mortalities. Evidence from our study suggests that the dose of leisure-time physical activity may modulate the cellular composition of peripheral blood, particularly with regard to neutrophil levels.

We observed an inverse association between leisure-time physical activity level and WBC count. Large observational studies have consistently reported that individuals performing more frequent or more intense physical activity, regardless of leisure-time or total physical activity, had a lower WBC count [17, 20, 22, 23, 28, 31, 32]. A higher objectively measured moderate-to-vigorous physical activity level was associated with a lower WBC count in the US adult population, healthy US male population, and US youth population in previous NHANES studies [17, 20, 31]. Another NHANES study observed that engaging in physical activity 22 or more times per month was associated with a 41% reduction in risk of a high WBC count (> 9.55 × 10^9^ cells/L), compared to engaging in activity less than four times per month [28]. Other large cohorts, including the British Regional Heart Study, the Cardiovascular Health Study, and the In CHIANTI study, also provided strong evidence for an inverse, dose–response association between physical activity and WBC count [22, 23, 32].

WBCs are actively involved in the inflammatory response through the release of cytokines, such as TNF α and IL-6 [4, 33]. These cytokines are inflammatory mediators that play an important role in the development of chronic diseases including CVD and cancer [1, 3]. In addition, WBCs are considered a predictive factor of CVD and cancer incidence: a higher WBC count is associated with a higher risk of coronary heart disease, stroke, lung cancer, breast cancer, colorectal cancer, and overall cancer [10–13, 34]. Our observation that a higher leisure-time physical activity level was associated with a lower WBC count in participants without CVD and cancer suggests that a physically active lifestyle may protect against low-grade inflammation in the healthy population, which may be important for the prevention of chronic diseases.

WBC itself is heterogeneous and comprises five immune cell populations. The major populations of WBCs are neutrophils (50–70%), followed in order by lymphocytes (20–40%), monocytes (2–8%), eosinophils (1–4%), and basophils (< 1%) [35]. Physical activity level or exercise training may affect each WBC population differently. The association of higher physical activity levels with a lower neutrophil count observed in our study was in agreement with previous studies [36, 37]. Neutrophils have been shown to be continuously recruited to the site of chronic inflammation through the release of serine proteases and the activation of other immune cells [6]. The observed lower neutrophil count associated with higher leisure-time physical activity levels might, therefore, suggest that leisure-time physical activity is associated with reduced levels of chronic inflammation. We observed that leisure-time physical activity level was inversely associated with monocyte count, but was not associated with lymphocyte count. However, a meta-analysis examining the effect of a combination of aerobic and resistance training (ranging from 4 weeks to 6 months) on WBC populations reported that exercise training significantly lowered lymphocyte count (pooled mean difference (95% CI) − 244 (− 475, − 13) cells/uL) for healthy adults, but had no effect on monocyte count (pooled mean difference (95% CI) 18 (− 18, 54) cells/uL) [36]. The contradictory observations for lymphocyte and monocyte counts across studies may be due to the fasting status, timing of blood collection, heterogeneity of study population, and types, duration, and intensity of physical activity. The associations between leisure-time physical activity level and lymphocytes and monocytes warrant further investigation.

Few studies have determined the effect of physical activity or long-term exercise training on platelet count. Our study observed that leisure-time physical activity level was not associated with platelet count in the total population. The observation was similar to a previous study that observed no association between moderate-to-vigorous physical activity and platelet counts [20]. However, we observed that the association between physical activity level and platelet count may differ by gender with an inverse association between leisure-time physical activity level and platelet count in men specifically. A similar, inverse dose–response relationship between physical activity level and platelet count was reported in a British male population [32]. One possible explanation for this gender-specific difference might be hormonal differences between males and females. Research suggests that the testosterone administration in men was associated with increases in platelet count [38], while estrogen may not have the same effect in women. Therefore, the impact of physical activity on platelet count may be influenced by sex hormones in different ways for males and females.

Platelets have recently been acknowledged as immune cells by some pathologists because of their participation in the immune response [39]. The role of platelets as immune cells in inflammation has also been extensively covered in a number of reviews [5, 39, 40]. Briefly, platelets are innate immune sensors that deal with infectious pathogens, initiate inflammation by producing an assortment of repair tools, such as cytokines and growth factors, and affect adaptive immune cells by interacting with WBCs. Future research evaluating the effect of exercise training not only on platelet count, but also on platelet function (e.g., platelet aggregation), may further elucidate the biological role of physical activity on immune function.

The inverse dose–response association between leisure-time physical activity level and NLR was first reported in our study. The lower NLR at higher leisure-time physical activity levels was mainly driven by the lower absolute count of neutrophils. A prior study reported that compared to no physical activity, participating in at least ten minutes of moderate or vigorous total physical activity at a time was associated with a lower NLR [19]. However, no dose–response association between increasing duration of moderate or vigorous total physical activity and NLR was found in that study [19]. The inconsistent observations suggest the potential for different biological roles of leisure-time physical activity versus total physical activity (the combination of leisure-time and non-leisure-time physical activity) on chronic inflammation. Notably, in a gender-specific analysis, leisure-time physical activity was inversely associated with NLR exclusively in women, not in men. While the exact reason for such a difference between gender is unknown, hormonal differences might be the issue. Estrogen, a predominant female hormone, has been shown to have anti-inflammatory properties [41]. Physical activity might interact with estrogen in a way that further enhances anti-inflammatory effects [42], resulting in a lower NLR in women. The associations of leisure-time physical activity level with PLR and LMR were first evaluated in our study. We did not observe associations between leisure-time physical activity and PLR or LMR. This indicated that NLR might be more sensitive to changes in leisure-time physical activity, than PLR and LMR.

Similar to WBC count, NLR, PLR, and LMR predict chronic inflammatory diseases such as CVD and cancer [8, 9, 14]. Research has shown that a higher NLR, a higher PLR, and a lower LMR are associated with poorer prognosis in cancer patients [43]. In addition, a higher NLR and a higher PLR are associated with a higher risk of all-cause mortality in the general US population [15, 44]. Knowing whether leisure-time physical activity level is associated with ratios of peripheral immune cell populations in the healthy population could provide biological evidence linking pre-diagnosis physical activity and future disease outcomes (incidence, prognosis, and mortality). Therefore, we assessed the association between leisure-time physical activity level and metrics of WBC count and NLR which predict mortality in the NHANES population. Compared to the lowest quartile of leisure-time physical activity (< 1.2 MET-hrs/wk), any physical activity above this would decrease the probability of WBC count above predictive thresholds for CVD and all-cause mortality (> 8.1 × 10^9^ cells/uL) as well as for cancer mortality (≥ 8.3 × 10^9^ cells/uL). This observation suggests that individuals with an inactive lifestyle may gain the greatest predictive benefit from small increases in leisure-time physical activity level, and that further increases in leisure-time physical activity level may result in diminishing returns. A recent meta-analysis provided evidence that the greatest risk reduction of CVD, cancer, and all-cause mortality was achieved from transitioning from sedentary to non-sedentary [45]. However, when assessing NLR > 2.68 as a predictive threshold for CVD and all-cause mortality, only participants with the highest leisure-time physical activity level displayed a significantly decreased probability of exceeding this NLR threshold. The highest quartile of leisure-time physical activity level (≥ 14 MET-hrs/wk) in our population is approximately equal to 3.5 h/wk (210 min/wk) of moderate exercise (e.g., brisk walking) or 1.75 h/wk (105 min/wk) of vigorous exercise (e.g., running or jogging). For reference, national guidelines recommend 2.5 h/wk (150 min/wk) of moderate exercise, or, 1.25 h/wk (75 min/wk) of vigorous exercise for adults [46].

The evidence base in exercise immunology supports a general consensus that the immune system is responsive to exercise. Evidence from our study supports the inverse association between leisure-time physical activity level and WBC count, neutrophil count, and NLR. Randomized controlled trials of exercise interventions have induced a response in WBC populations by decreasing WBC count, neutrophils, natural killer cells (a type of lymphocyte), and CD3 + T-cell (a type of lymphocyte) counts [37, 47, 48]. Not only does physical activity decrease the number of WBCs, but physical activity and exercise training also have demonstrated anti-inflammatory functional effects [16, 49]. Therefore, it is possible that the anti-inflammatory effect of leisure-time physical activity is a in part consequence of the observed association between leisure-time physical activity and WBC populations.

Our study has several strengths. First, we utilized a large nationally representative sample of the US population, which increases the generalizability of our results. Second, data collection in NHANES was conducted under robust quality assurance and control procedures. Standardized protocols and procedures for interviewer-administrated physical activity questionnaires and blood sample collection in NHANES have been extensively validated in previous studies [17, 19, 20]. Third, to the best of our knowledge, this is the first study assessing the association between leisure-time physical activity level and metrics of chronic inflammation which may predict mortality in the US general population.

The present study has several limitations. First, leisure-time physical activity level was self-reported. The information bias for physical activity level may be present because the duration and frequency of physical activity level may be difficult for participants to recall and quantify. Second, leisure-time physical activity level was quantified over a different length of time for NHANES cycles 1999–2006 (30 days) and NHANES cycles 2007–2018 (7 days). While cycle year was included in our models, and we conducted a sensitivity analysis, we cannot exclude heterogeneity from differences in questionnaires. Third, we excluded participants with a history or unknown history of diabetes, CVD, or cancers in the full sample, and adjusted for a history of arthritis as a confounder in the analysis model. Moreover, we observed a consistent observation with the full sample for a sensitivity analysis restricting to participants without a history of stroke, COPD, and hepatitis B. Nonetheless, confounding by unmeasured or poorly measured conditions that affect inflammation status is possible. However, we accounted for numerous established factors related to peripheral immune cell populations, many of which are validated, mitigating this concern. Fourth, temporal relationships could not be assessed due to the nature of cross-sectional study. Fifth, predictive thresholds of WBC count and NLR were based on old NHANES analyses, and not from a standard clinical reference range. Whether these thresholds are the best value for the current population as risk predictors needs to be further evaluated.

## Conclusions

In conclusion, a higher leisure-time physical activity level was associated with a lower WBC count and a lower NLR in our study. The decreased WBC count and NLR were mainly driven by decreased neutrophil counts, suggesting the future direction of investigating neutrophils related immune function within a range of physical activity levels. Any leisure-time physical activity level above the first quartile lowered the probability of having a detrimental WBC count (i.e. WBC level predictive of mortality outcomes). The highest leisure-time physical activity level was associated with a lower probability of a high NLR level that is predictive of increased risk for mortality. Leisure-time physical activity was not associated with LMR or PLR. Given the consideration that a lower WBC count and NLR predict a lower risk of CVD incidence, cancer incidence, CVD mortality, cancer mortality, and all-cause mortality, the findings of our study suggest that leisure-time physical activity level may be associated with low-grade inflammation that is important for future chronic disease outcomes.

### Supplementary Information

**Additional file 1: Table S1.** Adjusted means (95% CI) for peripheral immune cell populations by leisure-time physical activity level in men (*N* = 8363). **Table S2.** Adjusted means (95% CI) for peripheral immune cell populations by leisure-time physical activity level in women (*N* = 8730). **Table S3.** Adjusted means (95% CI) for peripheral immune cell populations and their ratios by leisure-time physical activity level in population without a history of stroke, COPD, and Hepatitis B (*N* = 16,183).

## Data Availability

The datasets used and/or analyzed during the current study are available at https://www.cdc.gov/nchs/nhanes/index.htm.

## Abbreviations

CBC: Complete blood counts
CI: Confidence interval
COPD: Chronic obstructive pulmonary disease
CVD: Cardiovascular disease
LMR: Lymphocyte-to-monocyte ratio
MEC: Mobile examination centers
MET-hrs/wk: Metabolic equivalent of task hours per week
NHANES: National Health and Nutrition Examination Survey
NLR: Neutrophil-to-lymphocyte ratio
OR: Odds ratio
PA: Physical activity
PIR: Family poverty income ratio
PLR: Platelet-to-lymphocyte ratio
WBC: White blood cell
