# Supplementary material for: Associations Between Leisure-Time Physical Activity Level and Peripheral Immune Cell Populations in the US General Population, Analysis of the National Health and Nutrition Examination Survey Data, 1999–2018
Source: Sports Med Open. 2023 Oct 28;9:101. doi: 10.1186/s40798-023-00643-y (PMC10613194; doi:10.1186/s40798-023-00643-y)
Supplement: Supplementary file 1 — Additional file 1: Table S1. Adjusted means (95% CI) for peripheral immune cell populations by leisure-time physical activity level in men (N = 8363). Table S2. Adjusted means (95% CI) for peripheral immune cell populations by leisure-time physical activity level in women (N = 8730). Table S3. Adjusted means (95% CI) for peripheral immune cell populations and their ratios by leisure-time physical activity level in population without a history of stroke, COPD, and Hepatitis B (N = 16,183). [file 40798_2023_643_MOESM1_ESM.docx]

**Title:** Associations between leisure-time physical activity and peripheral immune cell populations in the US general population, analysis of the National Health and Nutrition Examination Survey data, 1999-2018

**Journal:** Sports Medicine

**Authors:** Dan Lin, MPH^1^; Cheryl Thompson, PhD, MS^1^; Djibril M. Ba, PhD, MPH^1^; Joshua E. Muscat, PhD, MPH^1^; Shouhao Zhou, PhD^1^; Connie J. Rogers, PhD^2^; and Kathleen M. Sturgeon, PhD, MTR, MS^1^

^1^Department of Public Health Sciences, Pennsylvania State University College of Medicine, Hershey, PA 17033, USA

^2^Department of Nutritional Sciences, University of Georgia College of Family and Consumer Sciences, Athens, GA 30602, USA

**Corresponding Author:**

Kathleen M. Sturgeon, PhD, MEd, MS

Pennsylvania State University, College of Medicine

Penn State Cancer Institute, CH69

500 University Drive

Hershey, PA 17033

Telephone: (717)-531-4387

Fax: (717)-531-0580

Email: [kms99@psu.edu](mailto:kms99@psu.edu)

|  | **Leisure-time physical activity level, MET-hrs/wk** | | | |  |
| --- | --- | --- | --- | --- | --- |
|  | **Quartile1 (n=2,320)** | **Quartile2 (n=1,781)** | **Quartile3 (n=2,000)** | **Quartile4 (n=2,262)** | **P_linear_** |
| **WBC count, 1000 cells/uL, mean (95% CI)** | | | | | |
| Model 1^a^ | 6.94 (6.81, 7.06) | 6.54 (6.41, 6.67) | 6.59 (6.47, 6.71) | 6.53 (6.40, 6.66) | <0.001 |
| Model 2^b^ | 7.23 (6.87, 7.59) | 6.97 (6.60, 7.34) | 7.00 (6.65, 7.35) | 7.00 (6.63, 7.36) | 0.047 |
| **Neutrophil count, 1000 cells/uL, mean (95% CI)** | | | | | |
| Model 1^a^ | 4.07 (3.97, 4.18) | 3.80 (3.70, 3.91) | 3.82 (3.73, 3.92) | 3.77 (3.67, 3.87) | <0.001 |
| Model 2^b^ | 4.24 (3.96, 4.52) | 4.06 (3.79, 4.34) | 4.07 (3.81, 4.33) | 4.06 (3.80, 4.33) | 0.035 |
| **Lymphocyte count, 1000 cells/uL, mean (95% CI)** | | | | | |
| Model 1^a^ | 2.02 (1.98, 2.05) | 1.94 (1.90, 1.98) | 1.96 (1.92, 1.99) | 1.96 (1.91, 2.00) | 0.18 |
| Model 2^b^ | 2.15 (2.01, 2.28) | 2.11 (1.97, 2.24) | 2.11 (1.97, 2.25) | 2.12 (1.98, 2.26) | 0.63 |
| **Monocyte count, 1000 cells/uL, mean (95% CI)** | | | | | |
| Model 1^a^ | 0.58 (0.57, 0.59) | 0.56 (0.54, 0.58) | 0.56 (0.55, 0.58) | 0.56 (0.55, 0.57) | 0.045 |
| Model 2^b^ | 0.57 (0.54, 0.61) | 0.56 (0.51, 0.60) | 0.56 (0.53, 0.59) | 0.56 (0.52, 0.59) | 0.22 |
| **Platelet count, 1000 cells/uL, mean (95% CI)** | | | | | |
| Model 1^a^ | 245.16 (241.27, 249.06) | 240.54 (236.71, 244.37) | 239.51 (236.11, 242.91) | 238.36 (235.36, 241.37) | 0.02 |
| Model 2^b^ | 248.87 (241.96, 255.78) | 245.06 (238.01, 252.11) | 243.67 (236.88, 250.45) | 243.43 (237.14, 249.72) | 0.048 |
| **NLR, mean (95% CI)** | | | | | |
| Model 1^a^ | 2.17 (2.10, 2.24) | 2.08 (2.02, 2.15) | 2.09 (2.02, 2.15) | 2.07 (2.00, 2.13) | 0.049 |
| Model 2^b^ | 2.11 (1.93, 2.30) | 2.05 (1.87, 2.23) | 2.06 (1.87, 2.24) | 2.05 (1.88, 2.22) | 0.25 |
| **LMR, mean (95% CI)** | | | | | |
| Model 1^a^ | 3.69 (3.61, 3.77) | 3.71 (3.61, 3.81) | 3.70 (3.62, 3.79) | 3.72 (3.64, 3.80) | 0.66 |
| Model 2^b^ | 4.01 (3.74, 4.28) | 4.07 (3.79, 4.34) | 4.04 (3.77, 4.31) | 4.04 (3.77, 4.31) | 0.80 |
| **PLR, mean (95% CI)** | | | | | |
| Model 1^a^ | 131.82 (128.99, 134.65) | 133.52 (130.05, 136.99) | 131.81 (128.80, 134.82) | 132.16 (129.55, 134.78) | 0.85 |
| Model 2^b^ | 124.30 (116.52, 132.08) | 134.63 (126.14, 143.12) | 131.57 (123.02, 140.13) | 125.96 (118.02, 133.91) | 0.57 |

**Table S1.** Adjusted means (95% CI) for peripheral immune cell populations by leisure-time physical activity level in men (N=8,363).

WBC=white blood cell, MET=metabolic equivalent of task, CI=confidence interval, NLR=neutrophil-to-lymphocyte ratio, LMR=lymphocyte-to-monocyte ratio, PLR=platelet-to-lymphocyte ratio

^a^ Adjusted for age and survey year

^b^ Adjusted for age, survey year, BMI, race/ethnicity, family income, education level, marital status, smoking status, alcohol intake, daily energy intake, time of the blood draw, and history of arthritis

**Table S2.** Adjusted means (95% CI) for peripheral immune cell populations by leisure-time physical activity level in women (N=8,730).

|  | **Leisure-time physical activity level, MET-hrs/wk** | | | |  |
| --- | --- | --- | --- | --- | --- |
|  | **Quartile1 (n=2,560)** | **Quartile2 (n=1,941)** | **Quartile3 (n=2,055)** | **Quartile4 (n=2,174)** | **Plinear** |
| **WBC count, 1000 cells/uL, mean (95% CI)** | | | | | |
| Model 1a | 7.16 (7.04, 7.28) | 6.75 (6.62, 6.88) | 6.62 (6.51, 6.73) | 6.57 (6.44, 6.69) | <0.001 |
| Model 2b | 7.62 (7.23, 8.02) | 7.42 (7.03, 7.82) | 7.32 (6.92, 7.72) | 7.33 (6.94, 7.71) | 0.002 |
| **Neutrophil count, 1000 cells/uL, mean (95% CI)** | | | | | |
| Model 1a | 4.34 (4.24, 4.44) | 4.04 (3.94, 4.15) | 3.94 (3.84, 4.03) | 3.89 (3.78, 3.99) | <0.001 |
| Model 2b | 4.53 (4.20, 4.86) | 4.36 (4.05, 4.69) | 4.28 (3.93, 4.62) | 4.27 (3.94, 4.60) | 0.001 |
| **Lymphocyte count, 1000 cells/uL, mean (95% CI)** | | | | | |
| Model 1a | 2.08 (2.04, 2.11) | 2.00 (1.96, 2.04) | 1.98 (1.94, 2.01) | 1.97 (1.93, 2.01) | 0.003 |
| Model 2b | 2.30 (2.18, 2.42) | 2.29 (2.17, 2.41) | 2.27 (2.16, 2.39) | 2.29 (2.18, 2.40) | 0.77 |
| **Monocyte count, 1000 cells/uL, mean (95% CI)** | | | | | |
| Model 1a | 0.52 (0.51, 0.53) | 0.49 (0.48, 0.50) | 0.50 (0.49, 0.51) | 0.49 (0.48, 0.50) | <0.001 |
| Model 2b | 0.56 (0.53, 0.60) | 0.55 (0.51, 0.58) | 0.55 (0.51, 0.59) | 0.54 (0.51, 0.58) | 0.1 |
| **Platelet count, 1000 cells/uL, mean (95% CI)** | | | | | |
| Model 1a | 274.86 (270.67, 279.04) | 265.85 (261.14, 270.55) | 269.49 (264.69, 274.30) | 267.71 (263.80, 271.61) | 0.15 |
| Model 2b | 274.81 (268.00, 281.61) | 269.40 (262.00, 276.81) | 272.68 (265.58, 279.78) | 272.64 (265.63, 279.66) | 0.98 |
| **NLR, mean (95% CI)** | | | | | |
| Model 1a | 2.26 (2.18, 2.33) | 2.14 (2.08, 2.21) | 2.12 (2.05, 2.20) | 2.09 (2.03, 2.15) | 0.003 |
| Model 2b | 2.14 (1.90, 2.38) | 2.02 (1.79, 2.26) | 2.01 (1.76, 2.27) | 1.98 (1.74, 2.23) | 0.007 |
| **LMR, mean (95% CI)** | | | | | |
| Model 1a | 4.31 (4.21, 4.41) | 4.29 (4.18, 4.39) | 4.27 (4.17, 4.37) | 4.31 (4.19, 4.42) | 0.87 |
| Model 2b | 4.44 (4.12, 4.76) | 4.50 (4.17, 4.83) | 4.46 (4.12, 4.80 | 4.54 (4.25, 4.83) | 0.29 |
| **PLR, mean (95% CI)** | | | | | |
| Model 1a | 143.57 (140.77, 146.37) | 142.62 (139.46, 145.77) | 145.59 (142.58, 148.60) | 145.07 (141.86, 148.28) | 0.29 |
| Model 2b | 129.38 (120.89, 137.86) | 126.02 (117.29, 134.75) | 128.69 (120.17, 137.21) | 127.53 (118.79, 136.27) | 0.79 |

WBC=white blood cell, MET=metabolic equivalent of task, CI=confidence interval, NLR=neutrophil-to-lymphocyte ratio, LMR=lymphocyte-to-monocyte ratio, PLR=platelet-to-lymphocyte ratio

^a^ Adjusted for age and survey year.

^b^ Adjusted for age, survey year, BMI, race/ethnicity, family income, education level, marital status, smoking status, alcohol intake, menopausal status, female hormone use, daily energy intake, time of the blood draw, and history of arthritis.

**Table S3.** Adjusted means (95% CI) for peripheral immune cell populations and their ratios by leisure-time physical activity level in population without a history of stroke, COPD, and Hepatitis B (N=16,183).

|  | **Leisure-time physical activity level, MET-hrs/wk** | | | |  |
| --- | --- | --- | --- | --- | --- |
|  | **Quartile1 (n=4,638)** | **Quartile2 (n=3,565)** | **Quartile3 (n=3,891)** | **Quartile4 (n=4,089)** | **P_trend_** |
| **WBC count, 1000 cells/uL, mean (95% CI)** | | | | | |
| Model 1^a^ | 6.99 (6.89, 7.09) | 6.63 (6.52, 6.73) | 6.59 (6.51, 6.68) | 6.52 (6.43, 6.61) | <0.001 |
| Model 2^b^ | 7.34 (7.08, 7.60) | 7.14 (6.89, 7.40) | 7.11 (6.85, 7.37) | 7.11 (6.84, 7.38) | 0.001 |
| **Neutrophil count, 1000 cells/uL, mean (95% CI)** | | | | | |
| Model 1^a^ | 4.16 (4.08, 4.24) | 3.90 (3.82, 3.99) | 3.87 (3.79, 3.94) | 3.81 (3.74, 3.88) | <0.001 |
| Model 2^b^ | 4.34 (4.14, 4.55) | 4.19 (3.99, 4.39) | 4.16 (3.95, 4.36) | 4.15 (3.94, 4.36) | <0.001 |
| **Lymphocyte count, 1000 cells/uL, mean (95% CI)** | | | | | |
| Model 1^a^ | 2.04 (2.01, 2.07) | 1.97 (1.93,2.00) | 1.97 (1.94, 1.99) | 1.96 (1.93, 1.99) | 0.001 |
| Model 2^b^ | 2.20 (2.11, 2.28) | 2.17 (2.08, 2.25) | 2.17 (2.08, 2.25) | 2.17 (2.08, 2.25) | 0.32 |
| **Monocyte count, 1000 cells/uL, mean (95% CI)** | | | | | |
| Model 1^a^ | 0.55 (0.54, 0.56) | 0.53 (0.51, 0.54) | 0.53 (0.52, 0.54) | 0.52 (0.51, 0.53) | <0.001 |
| Model 2^b^ | 0.56 (0.53, 0.59) | 0.55 (0.52, 0.57) | 0.55 (0.52, 0.57) | 0.54 (0.52, 0.57) | 0.008 |
| **Platelet count, 1000 cells/uL, mean (95% CI)** | | | | | |
| Model 1^a^ | 258.62  (255.63, 261.60) | 253.52  (250.22, 256.81) | 254.54  (251.64, 257.44) | 252.94  (250.27, 255.67) | 0.043 |
| Model 2^b^ | 260.15  (253.79, 266.51) | 257.18  (250.82, 263.53) | 257.70  (251.58, 263.83) | 257.02  (250.80, 263.24) | 0.22 |
| **NLR, mean (95% CI)** | | | | | |
| Model 1^a^ | 2.19 (2.14, 2.34) | 2.11 (2.06, 2.16) | 2.09 (2.04, 2.14) | 2.08 (2.04, 2.12) | 0.003 |
| Model 2^b^ | 2.13 (1.99, 2.27) | 2.06 (1.92, 2.20) | 2.05 (1.90, 2.20) | 2.05 (1.91, 2.19) | 0.04 |
| **LMR, mean (95% CI)** | | | | | |
| Model 1^a^ | 4.01 (3.95, 4.07) | 4.00 (3.93, 4.08) | 4.00 (3.93, 4.08) | 4.00 (3.93, 4.07) | 0.85 |
| Model 2^b^ | 4.21 (3.99, 4.43) | 4.26 (4.03, 4.48) | 4.24 (4.02, 4.47) | 4.24 (4.03, 4.45) | 0.77 |
| **PLR, mean (95% CI)** | | | | | |
| Model 1^a^ | 136.91  (134.94, 138.88) | 138.27  (135.71, 140.83) | 138.53  (136.15, 140.91) | 139.15  (136.74, 141.56) | 0.16 |
| Model 2^b^ | 128.29  (122.94, 133.63) | 127.82  (121.67, 133.97) | 128.27  (122.10, 134.45) | 128.33  (122.63, 134.03) | 0.86 |

WBC=white blood cell, MET=metabolic equivalent of task, CI=confidence interval

^a^ Adjusted for age, gender, and survey year.

^b^ Adjusted for age, gender, survey year, BMI, race/ethnicity, family income, education level, marital status, smoking status, alcohol intake, daily energy intake, time of the blood draw, and history of arthritis.
